# Rare Xq23 Microdeletion Involving the DCX and PAK3 Genes Presenting With Lissencephaly, Agenesis of the Corpus Callosum, and Intractable Epilepsy: A Case Report

**DOI:** 10.7759/cureus.116506

**Published:** 2026-09-18

**Authors:** Dora H Safar, Zahraa F Attaa, Fatima Atieh, Raafat Hamad Seroor H Jadah

**Affiliations:** 1 Medicine and Surgery, Royal College of Surgeons in Ireland - Bahrain, Busaiteen, BHR; 2 School of Medicine, Royal College of Surgeons in Ireland - Bahrain, Busaiteen, BHR; 3 Pediatric Neurology, Bahrain Defence Force Hospital, Riffa, BHR

**Keywords:** agenesis of the corpus callosum, cnv analysis, dcx, epilepsy, global developmental delay, hemizygous deletion, lissencephaly, neuronal migration disorder, pak3, refractory seizures

## Abstract

X-linked lissencephaly spectrum disorder is a rare neurodevelopmental condition resulting from pathogenic variants that disrupt normal neuronal migration, with doublecortin (*DCX*) being one of the genes most frequently implicated. Similarly, pathogenic variants of the p21-activated kinase 3 (*PAK3*) gene are associated with X-linked syndromic intellectual disability, developmental delay, and epilepsy. Contiguous deletions involving both genes are exceedingly uncommon and may present with a complex neurological phenotype.

We report the case of a seven-month-old male with intellectual disability, global developmental delay, intractable epilepsy with generalized-onset seizures, lissencephaly, agenesis of the corpus callosum, hypertonia, hyperreflexia, gastroesophageal reflux, and constipation. Brain magnetic resonance imaging (MRI) demonstrated structural brain abnormalities, while whole exome sequencing (WES) with copy number variant (CNV) analysis identified a pathogenic hemizygous 258-kb deletion at Xq23 (110395597_110654202)x0), encompassing the *DCX* gene and partially involving the *PAK3* gene.

This report aims to expand the current limited understanding of the clinical manifestations associated with Xq23 microdeletions involving *DCX* and *PAK3*. It also highlights the importance of advanced genetic testing in children presenting with developmental delay, epilepsy, and structural brain abnormalities, allowing for early diagnosis, appropriate genetic counseling, and, ultimately, improved patient care.

## Introduction

Lissencephaly is a rare neuronal migration disorder characterized by abnormal cortical development, resulting in a smooth cerebral surface [1]. Pathogenic variants involving the doublecortin (*DCX*) gene are associated with X-linked lissencephaly spectrum disorders, which commonly present with developmental delay, intellectual disability, epilepsy, and structural brain abnormalities [2].

The p21-activated kinase 3 (*PAK3*) gene is another X-linked gene implicated in neurodevelopmental disorders and is associated with global developmental delay, language impairment, intellectual disability, epilepsy, and variable neuroimaging findings [3]. Deletions involving the adjacent genes *DCX* and *PAK3* are rare and may be associated with a broader clinical spectrum because multiple genes within the Xq23 region are affected [4].

Magnetic resonance imaging (MRI) often demonstrates characteristic abnormalities, including lissencephaly, pachygyria, subcortical band heterotopia (SBH), and corpus callosal defects [1]. Whole exome sequencing (WES) has become an important diagnostic tool in identifying genetic etiologies in children presenting with unexplained developmental delay, epilepsy, and abnormal neuroimaging findings [5].

## Case presentation

This is a seven-month-old boy, a known case of global developmental delay and epilepsy. He is the product of a full-term delivery, born at 38 weeks' gestation by spontaneous vaginal delivery, with Apgar scores of 9 and 10 at one and five minutes, respectively. His birth weight was 2.9 kg, with no antenatal or postnatal complications. The baby was born to first-degree cousins and has one female sibling who is alive and healthy. The patient was noted to have abnormal body movements shortly after birth. The parents described the episodes as body shaking associated with circumoral cyanosis and frothy mouth secretions, lasting for about one minute, followed by a postictal phase of drowsiness lasting about 30 minutes.

These episodes occurred multiple times during the day. There was no history of fever, vomiting, changes in bowel or urinary habits, or head trauma.

The patient was initially started on phenobarbital 10 mg twice daily (5 mg/kg/day). At eight weeks of age, the patient's weight was 4 kg. The antiepileptic medication was switched to levetiracetam, with a maintenance dose of 20 mg twice daily (10 mg/kg/day). The patient's epilepsy was controlled with levetiracetam until four seizure attacks occurred.

At the age of six months, the patient started to show significant motor and cognitive impairment in the form of poor eye contact with no social interaction, loss of the ability to roll over, and inability to maintain a seated position with support or reach for objects.

His physical examination showed delayed head control, poor social interaction, and no eye contact. No dysmorphic features were appreciated, and no neurocutaneous skin lesions were noted. Muscle tone was increased in all four limbs, with power of 4 out of 5 and deep tendon reflexes of +3, with no clonus or cerebellar signs. The remainder of the neurological examination was unremarkable.

His initial investigations showed abnormal electroencephalography (EEG) results due to the presence of left centro-temporal epileptiform spike-and-wave discharges, as shown in Figure 1.

**Figure 1:**
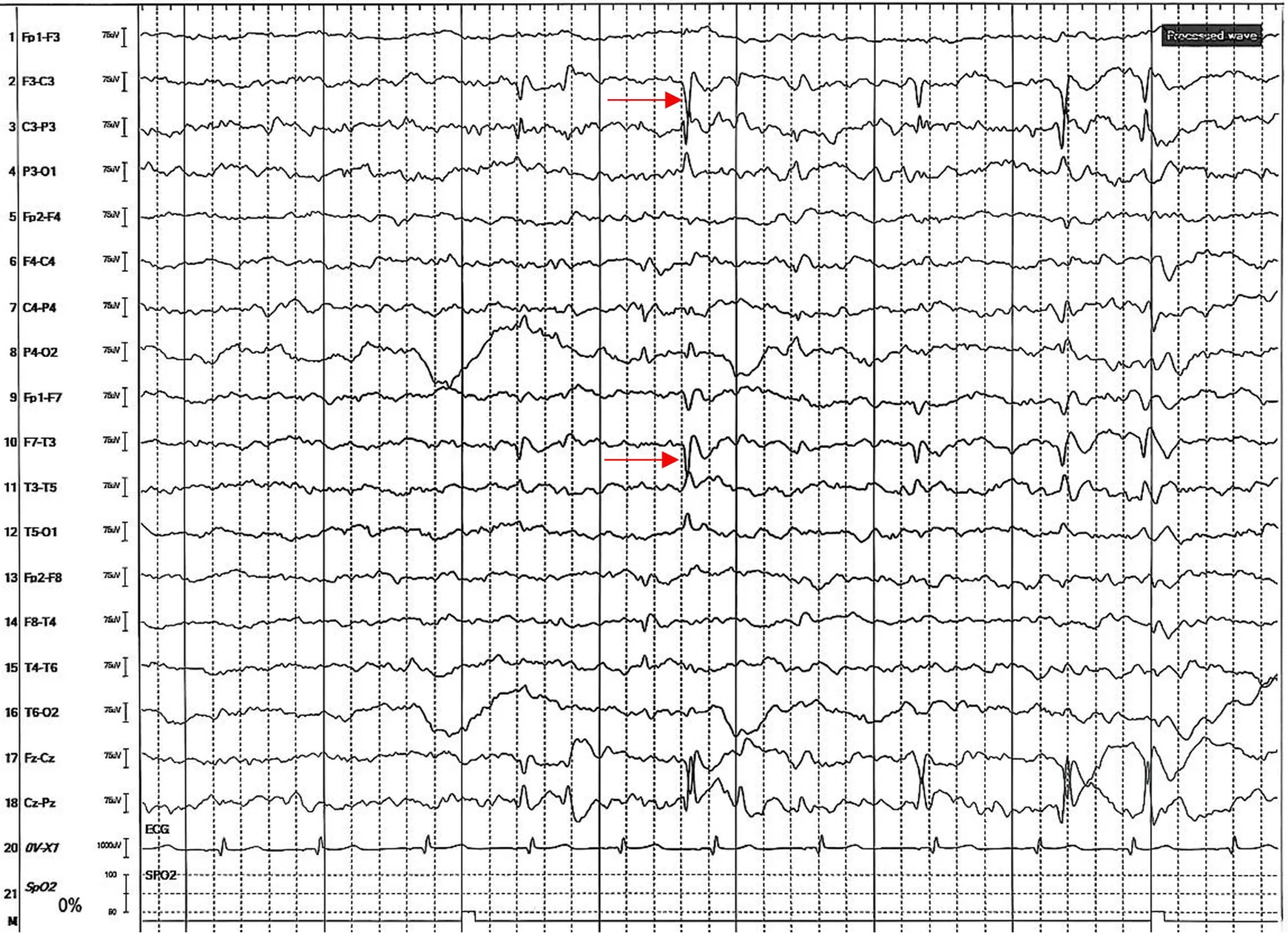
EEG demonstrating left centro-temporal epileptiform spike-and-wave discharges. Red arrows indicate the epileptiform spike-and-wave activity. EEG: Electroencephalography

His brain MRI was consistent with features of lissencephaly and agenesis of the corpus callosum. As seen in Figures 2-4, there is diffuse abnormality of the supratentorial mantle, with reduction of the gyri and marked thickening of the cortex.

**Figure 2:**
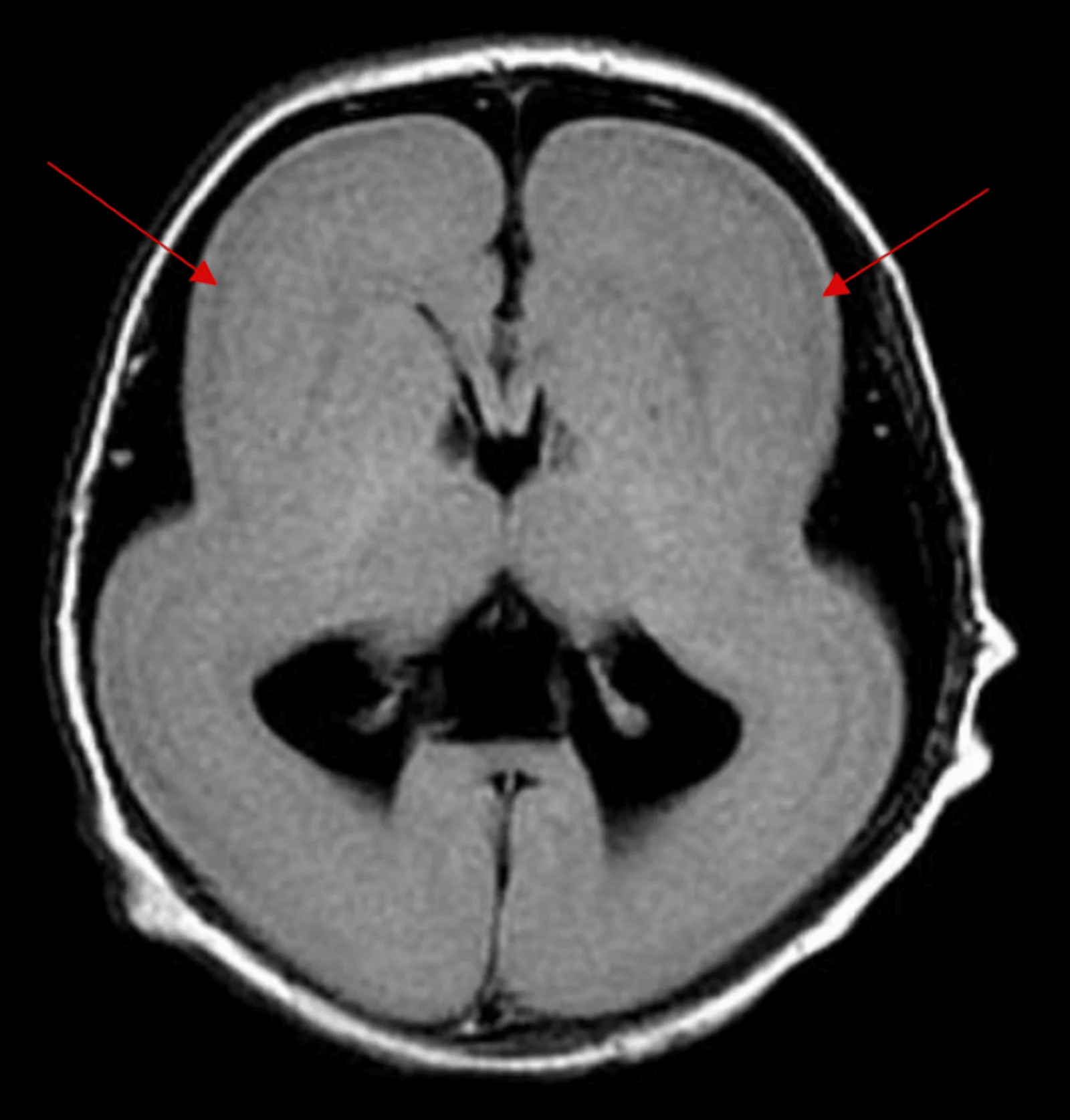
Brain MRI, axial FLAIR sequence demonstrating a smooth cerebral surface with marked reduction of the cerebral sulci and gyri, consistent with lissencephaly. Red arrows indicate the abnormally smooth cerebral cortex. MRI: Magnetic resonance imaging; FLAIR: Fluid-attenuated inversion recovery

**Figure 3:**
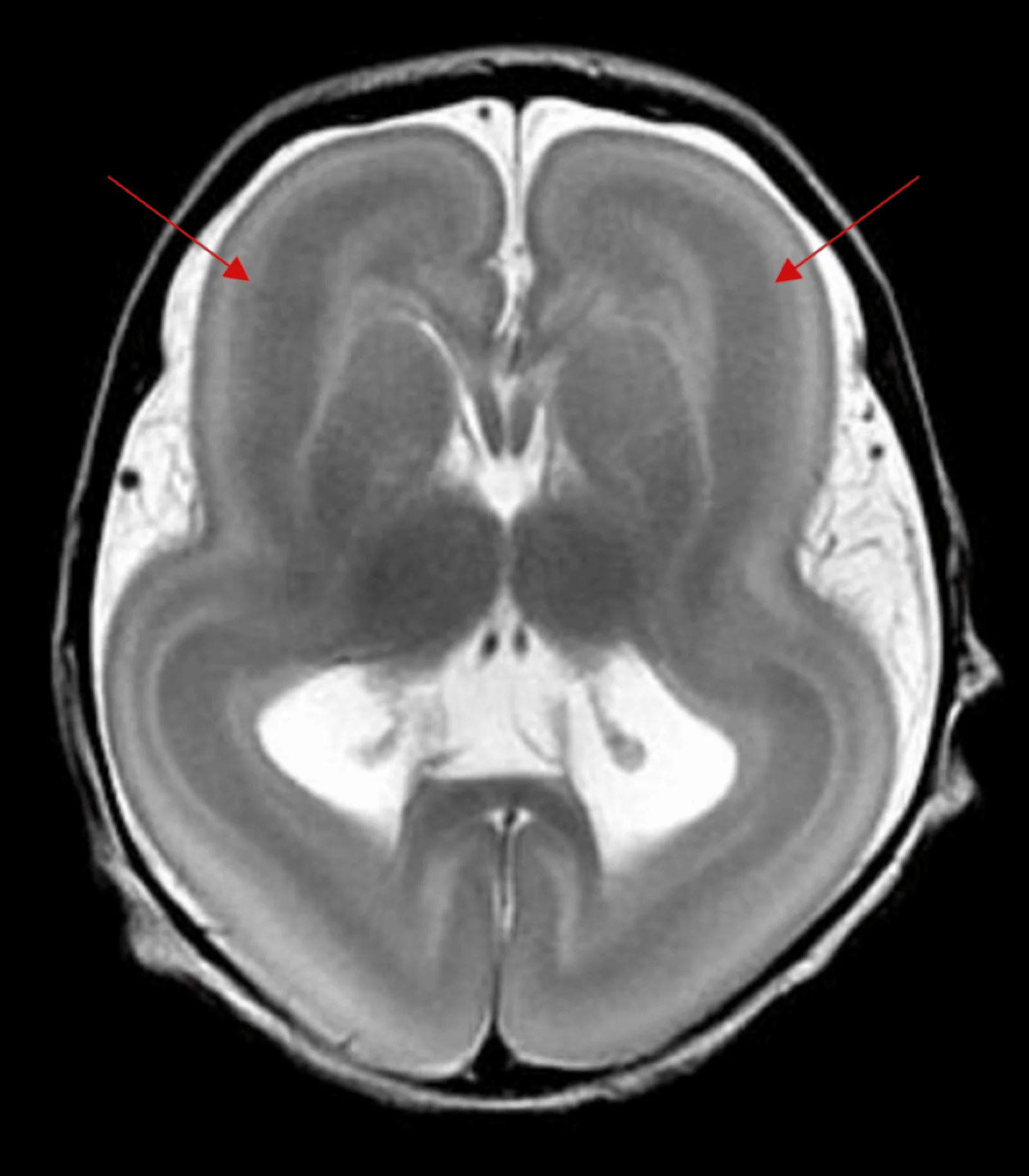
Brain MRI, axial T2-weighted sequence, demonstrating a smooth cerebral surface with marked reduction of the cerebral sulci and gyri, consistent with lissencephaly. Red arrows indicate the abnormally smooth cerebral cortex. MRI: Magnetic resonance imaging

**Figure 4:**
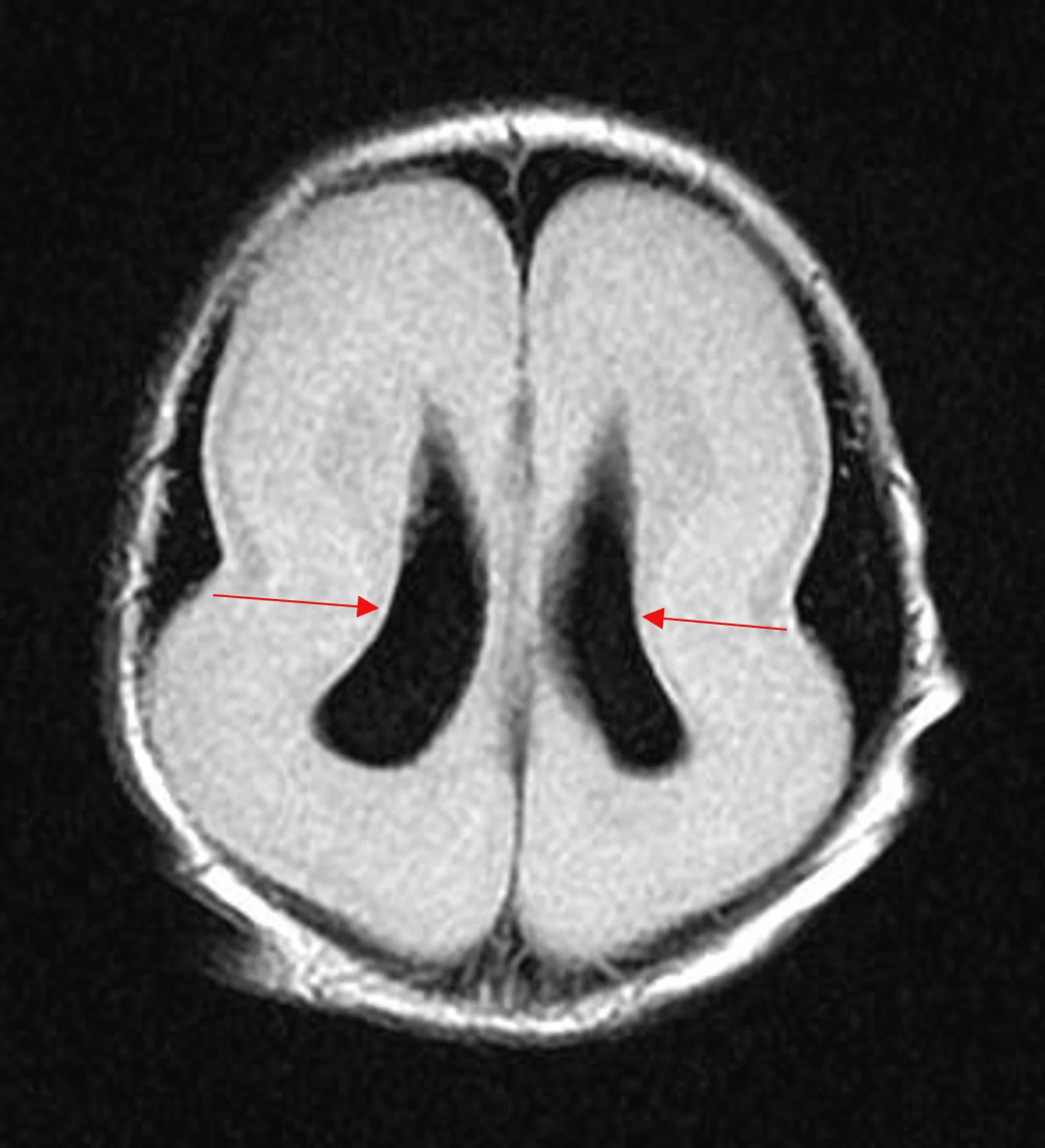
Brain MRI, axial FLAIR sequence demonstrating parallel-shaped lateral ventricles, consistent with agenesis of the corpus callosum. Red arrows indicate the parallel orientation of the lateral ventricles resulting from the absence of the corpus callosum. MRI: Magnetic resonance imaging; FLAIR: Fluid-attenuated inversion recovery

Based on the patient's clinical presentation, global developmental delay, and brain MRI findings, the patient underwent WES, which confirmed the diagnosis of X-linked lissencephaly spectrum disorder and X-linked syndromic intellectual disability, as shown in Table 1.

**Table 1:** WES with CNV analysis results. WES: Whole exome sequencing; CNV: Copy number variant; NGS: Next-generation sequencing; *DCX*: doublecortin; *PAK3*: p21-activated kinase 3

Positive result	Pathogenic CNV identified
Interpretation	NGS-based CNV analysis identified a loss of approximately 258 kb (copy number state: 0; classification: pathogenic) involving the Xq23 chromosomal region, including the *DCX* gene and partially involving the *PAK3* gene. This result is consistent with the genetic diagnoses of X-linked lissencephaly spectrum disorder and X-linked *PAK3*-related syndromic intellectual disability. No further clinically relevant variants related to the described phenotype were detected.

## Discussion

Disorders associated with the *DCX* and *PAK3* genes have been reported individually in the literature [2,3,6,7]. However, Xq23 microdeletions involving neurodevelopmental genes are rare [4]. Qin et al. reported a 671-kb Xq23 microdeletion involving AMMECR1 and CHRDL1 in a child with mild motor delay, growth retardation, midface hypoplasia, renal dysplasia, and cardiac and skeletal abnormalities [4]. In comparison, our patient had a smaller 258-kb Xq23 microdeletion involving *DCX* and partially involving *PAK3*, and presented predominantly with severe neurological manifestations, including global developmental delay, refractory epilepsy, lissencephaly, and agenesis of the corpus callosum. This comparison highlights the phenotypic variability associated with Xq23 microdeletions.

The *DCX* gene, located on chromosome Xq23, encodes doublecortin, a microtubule-associated protein that has a key role in neuronal migration during embryonic brain development [2]. Disruption of *DCX* function impairs the normal migration of cortical neurons, resulting in abnormal cortical lamination and the characteristic lissencephalic appearance [2]. In affected males, *DCX*-related disorders typically present with severe developmental delay, profound language and cognitive impairment, hypotonia, intellectual disability, and epilepsy [2,6,8,9]. The lissencephaly observed in this case aligns with *DCX*'s established role in cortical development and neuronal organization.

The *PAK3* gene, also located on the X chromosome, encodes p21-activated kinase 3, a protein involved in neuronal signaling, dendritic spine development, synaptic plasticity, and intellectual function [7,10]. Pathogenic variants in *PAK3* are associated with intellectual disability, global developmental delay, epilepsy, language impairment, and structural cerebral abnormalities [3,7]. The severe neurodevelopmental deficits observed in this case, including poor social interaction, impaired motor development, and developmental regression, may be attributable, at least in part, to the involvement of *PAK3*. The clinical manifestations observed in our patient overlap substantially with the phenotypic features reported in both *DCX*-related and *PAK3*-related neurodevelopmental disorders [2,3,7].

Taken together, the findings observed in our patient are consistent with the reported phenotypic spectrum of both *DCX*- and *PAK3*-related neurodevelopmental disorders. The coexistence of lissencephaly, severe developmental delay, epilepsy, and impaired motor and social development may reflect the combined involvement of these genes. Considerable genotype-phenotype variability has been described among individuals with Xq22.3-Xq23 microdeletions, reflecting differences in deletion size and gene content [4].

Neuroimaging plays a critical role in diagnosis, revealing characteristic findings that vary by gender, such as lissencephaly, pachygyria, SBH, and corpus callosum abnormalities [2]. Diagnosis of *DCX-PAK3*-associated neurodevelopmental disorders primarily depends on genetic testing, which is considered the gold standard for diagnosis [2]. WES, particularly when combined with copy number variant (CNV) analysis, is a valuable diagnostic tool for investigating the genetic basis of unexplained developmental delay, epilepsy, and abnormal neuroimaging findings, as it enables the detection of both sequence variants and chromosomal deletions [5].

The prognosis for patients with *DCX*-associated lissencephaly is primarily determined by the severity of cortical malformation, degree of developmental impairment, and presence of epilepsy [2,8,9]. While survival is possible, affected individuals frequently experience significant neurological morbidity and may develop complications such as feeding difficulties, aspiration, and recurrent respiratory infections [2,9]. Currently, no disease-modifying treatment exists for *DCX*-associated lissencephaly; management is therefore supportive and focuses on seizure control, prevention of complications, and optimization of quality of life [2,8]. These cases require a multidisciplinary approach, including pediatric neurology, rehabilitation services, nutritionists, and genetic counseling, which are essential for comprehensive long-term care [2,8].

In this case, the clinical presentation of early-onset epilepsy, severe developmental delay, and underlying cortical malformation suggests a guarded neurodevelopmental prognosis. The involvement of both *DCX* and *PAK3* may have contributed to the complex neurological phenotype observed in this patient. *DCX* plays a central role in neuronal migration and cortical organization, whereas *PAK3* is involved in neuronal signaling, dendritic spine development, and synaptic function [2,10]. Consequently, disruption of both genes may be associated with a broader spectrum of neurological manifestations than those typically observed with alterations affecting either gene individually. However, the rarity of such deletions limits definitive conclusions regarding their long-term clinical impact and highlights the need for additional case reports and longitudinal follow-up studies [4,11].

## Conclusions

Xq23 microdeletions involving the *DCX* and *PAK3* genes are rare genetic abnormalities associated with global developmental delay, epilepsy, and structural brain abnormalities. This case highlights the importance of considering an underlying genetic etiology in children presenting with unexplained developmental delay and refractory seizures.

WES, particularly when combined with CNV analysis, represents a valuable diagnostic approach that can facilitate early diagnosis, appropriate genetic counseling, and timely multidisciplinary management. Reporting rare cases such as this contributes to the expanding genotype-phenotype spectrum associated with Xq23 microdeletions and may improve understanding of their clinical manifestations and long-term outcomes.
